# A Pilot Randomized, Clinical Trial of the Anti-pruritus Effect of Melatonin in Patients with Chronic Liver Disease

**DOI:** 10.22037/ijpr.2020.112942.14024

**Published:** 2021

**Authors:** Ayda Esmaeili, Mohssen Nassiri Toosi, Mohammad Taher, Shahin Merat, Jaleh Bayani, Zahra fruzan. Karimian, Aysan Esmaeili, Bobak Moazzami, Soha Namazi

**Affiliations:** a *Research Center for Experimental and Applied Pharmaceutical Sciences, Urmia University of Medical Sciences, Urmia, Iran. *; b *Department of Clinical Pharmacy, School of Pharmacy, Urmia University of Medical Sciences, Urmia, Iran. *; c *Department of Internal Medicine, School of Medicine, Tehran University of Medical Sciences, Tehran, Iran. *; d *Liver* *Transplantation* *Research Center, Tehran University of Medical Sciences, Tehran, Iran. *; e *Digestive Disease Research Center, Shariati Hospital, Tehran University of Medical Sciences, Tehran, Iran. *; f *Department of Clinical Pharmacy, School of Pharmacy, Iran University of Medical Sciences, Tehran, Iran. *; g *Department of Internal Medicine, School of Medicine, Urmia University of Medical Sciences, Urmia, Iran. *; h *Department of Clinical Pharmacy, school of Pharmacy, Tehran University of Medical Sciences, Tehran, Iran. *

**Keywords:** Melatonin, Pruritus, Chronic liver disease, Visual analog scale, 12-Item pruritus severity score

## Abstract

Pruritus is one of the disturbing complications induced by chronic liver disease (CLD), bearing a negative impact on patient quality of life and potentially resulting in early liver transplants. Given the main role of the autotaxin enzyme in pruritus induced by CLD and the suppressive effects of melatonin on the expression of the autotaxin gene, this study was designed to evaluate the antipruritic effect of melatonin in patients with CLD. A double-blind, cross-over, randomized, placebo-controlled pilot trial was conducted on patients with CLD -induced pruritis. Patients were randomly assigned to two groups where they received melatonin 10-mg at night or placebo for 2 weeks. After a 2-week washout period, patients were then crossed over to the other group. The Visual Analog Scale (VAS) and the 12-Item Pruritus Severity Score (12-PSS) were used to assess patient response to therapy as the co-primary outcomes, while liver function tests were assayed too. Forty patients completed the study. The VAS score showed alleviation of 3.21 ± 2.24 (in pruritus) with melatonin (*p*-value <0.05). The study goal (a reduction of at least 20% in VAS) was achieved in 33 (82%) of study participants. In patients who received melatonin, the 12-PSS and Body Surface Area (BSA) affected by pruritus decreased on average 46.57% and 51.71%, respectively, with mood, sleep pattern and daily activity levels also demonstrating significant improvement (*p*-value < 0.05). Melatonin was found to be effective for managing pruritus in patients with CLD.

## Introduction

Pruritus is one of the main disturbing symptoms of Chronic Liver Disorders (CLD), such as primary biliary cholangitis (PBC), primary sclerosing cholangitis (PSC), chronic viral hepatitis, cirrhosis and drug-induced chronic cholestatic liver injury Garden, Ostrow and Roenigk (1, 2). The severity of pruritus can influence the patients’ quality of life and may lead to early liver transplants (3). Some hypotheses have been suggested for the causality of pruritus induced by CLD; however, the exact mechanism is unknown. The over-expression of the autotaxin enzyme leads to over-production of lysophosphatidic acid (LPA), which, similar to atopic dermatitis, has been known as an important itch mediator in patients with liver disease (4-7). 

Rifampicin is known as the most potent treatment for liver disease-induced pruritus; the most well-known mechanism has been described as an agonist of the Pregnane X receptor (*PXR*), which suppresses overexpression of the autotaxin enzyme (8). Nevertheless, rifampicin is not considered as the first-line therapy for this purpose due to possible side effects such as hepatotoxicity and high drug interaction potential (9, 10). Therefore, an investigation for determining an appropriate pharmacological therapy with the least side effects and drug interactions is ongoing.

Melatonin (N-acetyl-5-methoxytryptami-ne) is an endogenous hormone mainly secreted by the pineal gland into the plasma, which regulated circadian rhythm (11). Extrapineal melatonin was secreted by the hepatocytes and cholangiocytes into the gastrointestinal tract in magnitude amounts in compared to that which is formed in the pineal gland (12).

In previous animal and human studies, melatonin has shown hepatoprotective effects such as inhibiting bile duct hyperplasia, antifibrotic effect in fatty liver disease, reduction of liver enzymes, suppression of autotaxin gene expression and increase in antioxidant production (13-18). Furthermore, a decrease in melatonin levels in the GI tract was previously found in patients with PBC which is why a reduction in GI melatonin levels is thought to aggravate the liver disease (19), There are some similarities in the pattern and mechanism of pruritus induced by atopic dermatitis and liver diseases, such as overexpression of the autotaxin enzyme and elevated serum LPA levels. Given that several studies have shown that melatonin has had an antipruritic effect in patients with atopic dermatitis (20-22), it may also have a similar effect in patients with liver disease, which was presented as a hypothesis by Esmaeili *et al.* (23).

There are currently no published studies that have evaluated the impact of melatonin on pruritus in patients with liver disease. Therefore, the primary objective of this study was to assess the antipruritic effect of melatonin in patients with CLD.

## Experimental

*Methods*

*Study Design*

A double-blind, cross-over, randomized, placebo-controlled pilot study was conducted to investigate the antipruritic effect of melatonin in patients with CLD (18). Patients from the Liver Disease Clinic affiliated with Tehran University of Medical Sciences (TUMS) in Tehran, Iran, were recruited and enrolled in this clinical trial from July 15, 2018, to January 31, 2019. The study was approved by the Ethics Committee of TUMS (no. IR.TUMS.TIPS.REC.1397.043) and registered in the Iranian Registry of Clinical Trials (no. IRCT20180519039718N1). This study was conducted in accordance with the Helsinki declaration and followed all institutional and national guidelines, as well as regulations relevant to human experimentation (24). A written informed consent form was signed by all patients who agreed to participate in the study.

*Randomization and treatment phase*

A computer-generated randomization sequence was used to allocate eligible subjects in block sizes of four (A, B, C, D) within two groups: melatonin–placebo (MP) group or placebo–melatonin (PM) group. The statistician and study coordinator were the only individuals unblinded to the patient randomization schedule. The allocation sequences were concealed from the study investigators up until the study completion date. This study was conducted in 3 phases: In the first phase, patients were treated with the active drug administered orally as 2 pearls of melatonin 5 mg/day, given as 10 mg at night (the medication was produced by NutraLab Company, Canada and filled into pearls by Zahravi Pharmaceutical Companies, Iran), or placebo (with indiscernible shape, size, color and odor manufactured by Zahravi Pharmaceutical Companies, Iran) for 2 weeks. The second phase was a 2-week washout period. In the third phase, patients were crossed over to the other group (active drug or placebo, respectively) for another 2 weeks. The 10-mg daily dose of melatonin was chosen according to the study by Chojnacki et al. (25). During the study, any other antipruritic medications used by the patients were continued without any change, similar to before enrolling in the study. 

*Study participants*

Patients aged 18 years and older with pruritus lasting more than 4 weeks due to CLD (such as PBC, PSC, autoimmune hepatitis, chronic viral hepatitis, and prolonged drug-induced liver injury) were enrolled in the study. Participants from the following criteria were excluded: history of seizure, intolerance or history of hypersensitivity reaction to melatonin, prescribed any new antipruritic medication during the 4 weeks before their first visit, pregnancy, breastfeeding, decompensated liver disease, skin disease with pruritus (such as atopic dermatitis or eczema), unstable hemodynamic conditions, such as a mean arterial pressure <65 mmHg (26) or chronic kidney disease with creatinine clearance <15 mL/min or dialysis (27).

*Questionnaires and measurements*

The visual Analog Scale (VAS) and 12-item Pruritus Severity Score (12-PSS) questionnaires were used to assess patient response to therapy with melatonin (17, 18). Patient body surface area (BSA) affected by pruritus was calculated by using the figure in the Severity Scoring of Atopic Dermatitis Index (SCORAD) (16). Demographic characteristics (age and sex), basic clinical data including medical diagnosis, duration of itching, prior use of antipruritic medications and a history of allergies were collected. All eligible patients completed the VAS and 12-PSS questionnaires, marking the affected areas of their body on the figure. Baseline laboratory data, including Alanine Amino-transferase (ALT), Aspartate Aminotransferase (AST), Alkaline Phosphatase (ALP), total and direct bilirubin, Serum Creatinine (SCr), International Normalized Ratio (INR) for calculating the MELD score, Complete Blood Count (CBC) with differentiation and platelet count were assayed at the start and end of each treatment phase (thus, lab results were evaluated 4 times total during the study). Patients were asked to complete the VAS questionnaire every 3 days and the 12-PSS survey every 2 weeks up until trial completion. Social media applications (telegram and WhatsApp; no confidential patient information) as well as telephone calls/text messages were used to follow up with patients during each treatment phase and were referred to the Liver Disease Clinic every 2 weeks (following completion of each treatment phase).

*Statistical analysis*

A sample size of 32 subjects was calculated for a treatment effect (δ) of 2.37 and a power of 90% with 5% significance in order to detect at least 20% relief in VAS score by using Mayo *et al.* study (15). A total of 40 patients were enrolled in the study to take into account for a predicted 20% dropout rate. Medication compliance was defined as at least 80% adherence to therapy (11).

The statistical analysis in this study was carried out by using the SPSS software (version 20.0, SPSS Inc., Chicago, IL, USA). Continuous data were reported as mean ± SD, and categorical values were presented by frequency (percentage) and/or median (interquartile range). The Kolmogorov–Smirnov test was performed on numerical variables for evaluating the normality distribution. An independent t-test and Mann-Whitney U test were used to compare parametric and nonparametric variables, respectively. 

In considering the study design, mixed effect ANOVA was calculated using STATA version 13 (StataCorp, College Station, TX, USA) to evaluate the “treatment effect” of melatonin with omitting carryover and period effect. The number needed to treat (NNT) and 95% Confidence Interval (95% CI) for Cohen’s d were also calculated (28).

The VAS score was used for estimating the severity of pruritus based on perceptive symptoms and classified into fourth groups: mild (1-3), moderate (4-6), severe (7-8) and very severe ( >8) (17). In this study, since we did not have relatively equal numbers of patients in all four groups, patients were divided into two larger groups in order to evaluate the effect of baseline pruritis severity (based on VAS score) on patient response to therapy with melatonin: mild to moderate (0-6 VAS score) and severe to very severe (7-10 VAS score). 

*12-PSS*

Some instruments have been designed to evaluating pruritus with such as the 5-D itch scale, 12-PSS which were contain some questions evaluating mood, severity, extent and intensity (29).

The 12-PSS questionnaire included 12 questions, categorized into 5 main domains as follows: frequency of pruritus (question number 1), mood and daily activity (questions number 2-5), scratching intensity (questions number 6-8 and 12), the severity of pruritus (questions number 9-10) and extent of pruritus (question number 11) (18). 

Due to the lack of an existing translation of the 12-PSS questionnaire in the Persian language, this questionnaire was translated by 2 translators; then the validity and reliability of the approved Persian questionnaire were evaluated (Cronbach 𝛼 coefficient: 0.89) according to Beaton’s intercultural principles (30). The Wilcoxon test was used for evaluating the effect of melatonin on each domain and the correlation of VAS with 12-PSS was evaluated using the Pearson correlation test.

Anti-pruritus responses were compared between patients with cholestatic and non-cholestatic liver disease.

The Naranjo Adverse Drug Reaction (ADR) Probability Scale was applied to evaluate melatonin- and placebo-induced ADR in the study population (31). A *p*-value of <0.05 was considered as significant. 

## Results

*Patient characteristics*

A total of 49 patients were deemed eligible to enroll in the study. During the treatment period, 9 were lost to follow-up (Consort Flow Diagram 1). Finally, 22 patients were assigned to the PM group and 18 patients to the MP group, all of whom completed the study. Patient characteristics and diagnosis information are summarized in Table 1. There were no significant differences in age, gender, and distribution of pruritus intensity between the two groups (*p*-value > 0.05), whereas patient BSA affected by pruritus was different at baseline (*p*-value = 0.05). 

*Treatment response*

*Antipruritic effects*

A mixed-effects model of variance analysis revealed that treatment effects on VAS, 12-PSS and BSA were significant (*p*-value < 0.05). Carryover and period effect on VAS, 12-PSS and BSA are presented in Table 2.

Study outcomes (VAS, 12-PSS and area) were not influenced by sex (*p*-value > 0.05). The amount of decrease in VAS, 12-PSS and area with melatonin versus placebo at the end of each treatment phase was significant (*p*-value < 0.05) (Table 3). Thirty-three patients (82.50%) achieved the goal of antipruritic effect in their VAS score, and 24 patients (60%) had at least a 50% relief. 

The effect size (Cohen’s d) was 1.32 (CI 95%, 2.01-0.64) and the number needed to treat (NNT) was 1.90. There was no statistically significant difference in the amount of antipruritic response in patients with mild to moderate baseline VAS compared to those with a severe to very severe baseline VAS score (*p*-value = 0.53).

In comparing the response of patients who had taken antipruritic medications prior to enrolling in this study to those who had not received any prior antipruritic treatment, no significant differences were noted in the VAS, 12-PSS and BSA (*p*-values: 0.32, 0.91 and 0.95, respectively). The comparison of treatment response to melatonin in cholestatic patients (N:23) and non-cholestatic patients (N:17) also revealed no statistically significant difference between patients in the VAS score, 12-PSS, and BSA (*p*-value: 0.18, 0.41 and 0.99 respectively).

The Pearson Correlation analysis showed that VAS and 12-PSS had a strong correlation (r = 0.89, *p*-value < 0.001). The trend of changes in the average VAS score of patients on melatonin and/or placebo are illustrated in Figure S1 (in supplementary file). 

The antipruritic effect of melatonin on each of the five domains of the 12-PSS questionnaire was statistically significant (*p*-value = 0.001). The frequency and duration of itching decreased in 20 (50%) patients by at least 50%. The raw points from the questions related to the patient’s mood and daily activities improved significantly (*p*-value < 0.05) with melatonin in comparison to placebo (52.50% versus 1.66%). The frequency of nighttime awakenings declined for 62.50% of patients on melatonin (n = 25). Sleep disturbance improved by an average of 46.66% ± 7.30% points in patients on melatonin (*p*-value < 0.05).

The scratching intensity score showed a statistically significant alleviation (*p*-value <0.05) with melatonin in comparison to placebo (an average of 32.50% ± 40.28% versus 8.33% ± 49.45%). Sixteen (55.18%) of the 29 patients on melatonin who had scratch lesions on their skin at the beginning of the study experienced resolution of lesions, while just one (5.00%) of the 22 patients on placebo observed an improvement in excoriation (*p*-value < 0.05). The analysis of questions related to the extension of pruritis in the 12-PSS showed that 23 (57.5%) of 40 patients had a reduction in BSA affected by itching while on melatonin.

Liver function test results during the study were shown in Table 4.

Adverse outcomes: Four patients on melatonin failed to complete the study due to adverse drug reactions (ADRs), including GI disturbance (1 case), annoying headache (1 case) and drowsiness (2 cases). The Naranjo scale for all ADRs was probable. Five cases on placebo complained about GI upset, but only 2 of them were not able to complete the study. The Naranjo scale for 2 of the ADRs was probable while the remaining were possible. Patients who experienced ADRs were recommended not to take pearls on an empty stomach. Also, three of the patients on placebo had poor adherence to the study protocol, leading to their early drop out from the trial. 

## Discussion

Given melatonin’s suppression of the autotaxin gene expression alongside its pleiotropic effects in the liver and antipruritic effect in atopic dermatitis, we designed a pilot clinical trial to assess its effects on pruritus associated with CLD. Based on our search in PubMed and Scopus, there were no published animal or human studies on this topic. Therefore, the results of this study were not directly comparable to similar studies.

Some instruments, such as the 5-D itch scale, 12-PSS were designed to evaluate pruritus with different aspects (29). 12-PSS is a multidimensional tool same as 5-D itch scale for assessing different aspects of pruritus. 12-PSS were used in this study because its questions were easy to answer than 5-D itch scale to answer while containing all aspects assessed in the 5 –D itch scale.

*Antipruritic effects*

This study showed that melatonin had an antipruritic effect which resulted in a significant decrease in itching intensity, severity, extension and duration. The results were not related to the patient’s sex, the baseline severity of pruritus or history of using antipruritic medications prior to enrollment in the study. The reduction in VAS and 12-PSS scores with melatonin was significantly different compared to the placebo group (*p*-value < 0.05). We observed that melatonin alleviated the pruritic VAS score by 3.21 ± 2.24, with 33 (82.50%) patients achieving the study goal (a 20% reduction in VAS score) and 24 (60%) of patients having experienced at least a 50% improvement in itching. The average decrease in VAS and 12-PSS scores with melatonin was 46.35% and 46.57%, respectively, while the same scores with placebo were noted to have increased by an average of 11.69% and 4.56%, respectively. 

Moreover, no statistically significant difference was noted between cholestatic and non-cholestatic patients in the antipruritic response to treatment with melatonin (2.75 ± 2.09 versus 4.18 ± 2.28, *p*-value: 0.18).

As there were no similar trials in the literature that evaluated the effect of melatonin on pruritus associated with liver disease, we used the results from other antipruritic agents from similar studies to compare with ours. According to a meta-analysis in 2006 on placebo-controlled studies of rifampin used for treating pruritus associated with chronic cholestasis, 47 of 61 patients (77%) on rifampin had an acceptable antipruritic response (8). In this study, 33 of the 40 patients (82.5%) who received melatonin had an appropriate antipruritic response. 

The results of the current study were also superior to a study that investigated the use of sertraline (75-100 mg/day) for 6 weeks in 12 cholestatic patients. The average alleviation in the raw point and percentage of VAS score in that trial was 1.86 and 33%, respectively, and an acceptable response in VAS score ( 20% reduction) was observed in 66.67% of patients (32).

In the placebo-controlled study of bezafibrate 400 mg QID in 84 patients with PSC or PBC, 36% of patients achieved the study goal, which was defined as a 50% reduction in VAS score (33). In our study, 24 patients (60%) achieved at least a 50% reduction in VAS score.

The correlation between the VAS score and 12-PSS was strong (r = 0.89, *p*-value < 0.001). In the study by Reich and colleagues in 2017 which was conducted on 148 patients with chronic dermatological pruritus (more than 6-weeks), the correlation was reported to be strong with r = 0.58 (18). This finding was confirmed in our study.

As illustrated in Figure S1, the trend of antipruritic effect in the melatonin group had a linear pattern with a slope of -0.67 and R^2^: 0.96. The onset of antipruritic response to melatonin started with the first doses and also observed that the effect of melatonin did not reach a steady-state. Therefore, we concluded that melatonin might need longer than 2 weeks to reach the peak or optimum antipruritic effect.

It is worth noting that relief in the severity of itching should be accompanied by a concurrent decrease in the area affected by pruritus. In our study, the itching BSA was significantly decreased with melatonin versus placebo. (76.77% to 38.30% versus 63.02% to 62.12%, *p*-value < 0.05). This factor was not reported in other antipruritic agents’ studies. (24, 32 and 34).

The effect size (Cohen’s d) of 1.32 showed that melatonin had a large treatment effect in comparison with placebo in patients with CLD. Additionally, the NNT of 1.90 denoted that an adequate response to therapy is expected in at least one out of 2 patients on melatonin. 

Based on our study’s outcomes and a comparison with the findings from the above-mentioned studies (8, 32, 33), melatonin can be recommended as an efficient antipruritic agent in patients with CLD. Nevertheless, more studies with larger sample sizes conducted over longer time periods will be needed to confirm these results further.

The most common side effect was observed by melatonin was drowsiness, which was mentioned by Farrokhian et al. both studies reported one case with unusual headache (35).

Effects on sleep pattern, daily activity levels and mood 

Sleep disturbances due to pruritus could influence patient quality of life (2). Melatonin is known as an efficient medication to improve the onset, quality and duration of sleep (33). In this study, melatonin resulted in a statistically significant decrease of 46.66% in sleep disturbance episodes (waking up) during the night (*p*-value < 0.05) in comparison to placebo (8.33%). One explanation for this observation may be that melatonin alleviated intense itching during the night. Therefore, prescribing a medication that could target both sleep pattern and pruritus would be dually favorable to improving sleep hygiene. The same results were observed with naltrexone and rifampin studies; however, despite the antipruritic property of sertraline, improvement in sleep pattern was not observed (24, 32, 34).

The patients’ mood and daily activity significantly improved with melatonin compared to placebo (*p*-value < 0.05). This positive effect on mood and activity levels was similar to sertraline, an antidepressant agent (32).

**Table 1:** Patient characteristics and diagnosis data at baseline

**Group (n = 40)**	**Melatonin-placebo** **(n = 18)**	**Placebo-melatonin** **(n= 22)**	***p*** **-value** ^*^
Age, year, mean ± SD	41.5 ± 12.08	49.73 ± 12.89	0.2
Sex (F/M)	7/11	11/11	0.48
**Etiology** ^§^			
	
PSC, PBC, drug induced Liver disease (cholestatic)	10	13	
Cirrhosis ( induced by HBV, HCV, AIH, idiosyncratic) (non-cholestatic)	8	9	
VAS base			0.87
Mild (≤3)	1 (5.60%)	0 (0.0%)	
Moderate (4-6)	6 (33.30%)	8 (36.4%)	
Sever (7-8)	7 (38.90%)	10 (45.5%)	
Very sever (9-10)	4 (22.20%)	4 (18.2%)	
VAS base, mean ± SD	7.28 (1.81)	7.50 (1.60)	0.68
12-PSS base, mean ± SD	15.28 ± 3.88	15 ± 4.48	0.84
BSA, median (Q1-Q3)	84.75 (61.75-95)	81 (57.35-95)	0.05
**Baseline laboratory data, mean ±SD**			
ALT, U/L	82.53 ±75.08	79.20 ± 75.57	0.84
AST, U/L	73.30 ±52.43	81.86 ± 63.87	0.57
ALP, U/L	738.64 ± 593.77	575.36 ± 431.44	0.15
Bilirubin Total, mg/dL, Median (Q1-Q3)	2.85 (1.32 -8.60)	1.3 (0.87-3.75)	0.01
Bilirubin direct, mg/dL, Median (Q1-Q3)	1.3(0.66-6.72)	0.48(0.3-1.75)	<0.05
PLT × 10^3^/mm^3^	195.17 ± 104.65	168.41 ± 86.00	0.21
INR, Median (Q1-Q3)	1.07 (0.93-2)	1.11 (1-2.7)	0.24
MELD	11.25 ± 6.04	9.93 ± 4.82	0.28
**Anti-pruritus treatment ** **(patient no)**			
			0.75
Doxepin, Sertraline	5	5	
Hydroxyzine, cetirizine	2	2	
Rifampin	1	1	
Cholestyramin	1	1	
Naltrexone	1	0	

PBC: primary biliary cholangitis; PSC: primary sclerosing cholangitis; HBV: Hepatitis B virus; HCV: Hepatitis C virus; AIH: autoimmune hepatitis; HCC*: *hepatocellular carcinoma; ALT: Alanine transaminase; AST: Aspartate transaminase; ALP: Alkaline phosphatase; INR: International normalized ratio; PLT: Platelets; MELD Score: Model For End-Stage Liver Disease; SCr: Serum Creatinine; VAS: Visual analog scale; 12-PSS: 12-item pruritus severity score; BSA: body surface area; n: number of patient. *p-value < 0.05 is significant. ^§^There were 11 patients with overlapping etiologies (eg; AIH/cirrhosis). (47.5% PSC, 42.5% Cirrhosis, 5% PBC, 12.5% AIH, 7.5% HBV and 7.5% HCV).

**Table 2:** Mixed effects model of variance analysis

***p*** **-value** ^*^			
**period effect**	**Carry-over effect**	**Treatment effect**	
0.30	<0.001	<0.001	VAS
0.12	0.06	<0.001	12-PSS
0.36	0.09	<0.001	Area

VAS: Visual analog scale; 12-PSS: 12-item pruritus severity score; Area: body surface area.

*p*-value < 0.05 is significant. ^*^p-value after treatment versus baseline.

**Table 3:** Comparing anti-pruritus effect of melatonin and placebo on VAS, 12-PSS and area in all patients and intra-groups

**Total (n = 40)**				**PM group (n = 22)**				**MP group (n = 18)**			
***p*** **-value**	**P**	**M**		***p*** **-value**	**P**	**M**		***p*** **-value**	**P**	**M**	
											**VAS**
0.07	5.85 ± 2.29	6.80 ± 2.03		0.81	6.54 ± 2.58	6.73 ± 2.21		0.01	5.00 ± 2.46	6.89 ± 1.71	before each phase
0.10	5.65 ± 2.25	3.59 ± 2.17		0.08	5.95 ± 2.50	3.86 ± 2.27		0.76	5.28 ± 2.61	3.25 ± 1.91	after each phase
<0.001	0.20 ± 2.29	3.21 ± 2.24		0.01	0.59 ± 2.10	2.86 ± 2.21		<0.001	-0.28 ± 1.90	3.64 ± 2.26	difference of VAS
<0.001	-11.69% ± 60.87	46.35% ± 26.35		0.054	-1.41% ± 53.88	41.3% ± 27.03		<0.001	-24.25% ± 67.90	52.42 ± 25.03	Mean percent decrease^†^
	0.58	<0.001			0.20	<0.001			0.64	<0.001	*p*-value^*^
							**12-PSS**				
0.03	12.37 ± 5.23	14.26 ± 4.24		0.56	14.95 ± 4.42	14.09 ± 4.52		<0.001	9.22 ± 4.43	15.27 ± 3.88	before each phase
<0.001	11.87 ± 4.80	7.97 ± 4.52		0.02	12.59 ± 4.94	9.05 ± 5.05		0.003	11.00 ± 4.60	6.66 ± 3.48	after each phase
<0.001	0.50 ± 3.54	6.65 ± 3.75		0.004	2.36 ± 2.68	5.04 ± 3.17		<0.001	-1.78 ± 3.15	8.61 ± 3.07	difference of 12-PSS
<0.001	-4.56%	46.5%		0.001	15.7%	37.9%		<0.001	-29.36%	51.10%	Mean percent of decrease
	0.37	<0.001			<0.001	<0.001			0.03^§^	< 0.001	*p*-value^*^
							**Area**				
0.02	63.02 ± 26.30	76.77 ± 21.77		0.65	76.54 ± 22.02	72.88 ± 23.30		<0.001	46.50 ± 21.50	81.52 ± 19.28	before each phase
<0.001	62.12 ± 21.64	38.30 ± 22.83		0.001	66.80 ± 21.08	41.09 ± 26.00		0.002	52.42 ± 21.54	34.67 ± 17.25	Area after each phase
<0.001	0.90 ± 18.48	38.57 ± 19.86		<0.001	9.75 ± 14.82	31.80 ± 20.84		<0.001	-9.92 ± 16.94	46.86 ± 17.25	difference of area
<0.001	-10.65	51.71		<0.001	11.31	45.98		<0.001	-37.48	58.70	Mean percent of decrease
	0.76	<0.001			0.006	<0.001			0.02	<0.001	*p*-value^*^

*p*-value < 0.05 is significant.

All data were presented in mean ± SD.

*p-value of difference intra group.

§ *p*-value 0.03 due to negative effect.

MP group: melatonin-placebo; PM group: placebo-melatonin; 12-PSS: 12-item pruritus severity score, VAS: Visual analog scale, area: body surface area.

^†^Mean percent of decrease: (difference of parameter after and before each phase/before each phase) × 100.

**Table 4:** Comparing melatonin and placebo effects on liver enzyme and function tests after 2 weeks

**placebo**	**melatonin**		
77.07 ± 72.33	84.32 ± 78.11	At base line	ALT (U/L)
70.42 ± 41.71	59.20 ± 37.86	After treatment	
6.65 ± 63.55	25.12 ± 56.82	Difference of base and after treatment	
0.51	0.008	*p*-value^*^	
74.55 ± 57.52	82.37 ± 60.44	At base line	AST (U/L)
73.82 ± 47.59	68.57 ± 44.21	After treatment	
0.72 ± 29.56	13.80 ± 39.05	Difference of base and after treatment	
0.87	0.03	*p*-value^*^	
650.50 ± 493.10	642.17 ± 540.30	At base line	ALP (U/L)
624.18 ± 450.93	592.50 ± 454.50	After treatment a	
31.32 ± 263.43	49.67 ± 231.30	Difference of base and after treatment	
0.45	0.18	*p*-value^*^	
3.66 ± 6.42	5.23 ± 10.03	At base line	Bilirubin Total (mg/dL)
3.85 ± 6.17	4.22 ± 7.59	After treatment	
-0.30 ± 1.70	1.01 ± 3.63	Difference of base and after treatment	
0.18	0.01	*p*-value^*^	
2.06 ± 3.75	3.14 ± 5.31	At base line	Bilirubin Direct (mg/dL)
2.29 ± 4.05	2.52 ± 4.7	After treatment	
-0.22 ± 1.14	0.61 ± 2.15	Difference of base and after treatment	
0.21	0.01	*p*-value^*^	
1.25 ± 0.40	1.29 ± 0.37	At base line	INR
1.23 ± 0.35	1.24 ± 0.38	After treatment	
0.01 ± 0.20	0.05 ± 0.24	Difference of base and after treatment	
0.80	0.15	*p*-value^*^	
185.22 ± 98.39	175.67 ± 92.84	At base line	PLT × 10^3^/mm^3^
176.45 ± 91.45	191.10 ± 98.82	After treatment	
8.77 ± 25.80	-15.42 ± 24.99	Difference of base and after treatment	
0.03^†^	<0.001	*p*-value^*^	
0.88(0.24)	0.90(0.23)	At base line	SrCr mg/dL
0.89(0.23)	0.86(0.23)	After treatment	
-0.01(0.09)	0.03(0.13)	Difference of base and after treatment	
0.77	0.10	*p*-value^*^	
9.87 ± 5.17	11.17 ± 5.62	At base line	MELD score
10.17 ± 5.37	9.77 ± 5.27	After treatment	
-0.30 ± 1.69	1.40 ± 2.89	Difference of base and after treatment	
0.27	0.004	*p*-value^*^	

ALT: Alanine transaminase; AST: Aspartate transaminase; ALP: Alkaline phosphatase; INR: International normalized ratio; PLT: Platelets; SrCr: serum creatinine; MELD Score: Model For End-Stage Liver Disease.

All data were presented in mean ± SD.

*p*-value < 0.05 is significant.

* ***p*** **-value** **of difference intra group.**

**†** ***p*** **-value** **0.03 due to negative effect.**

## Limitations

The analysis of our study’s data showed a significant carryover effect (*p*-value <0.05) with a 2-week washout period following a 10-mg once-daily dose of melatonin, indicating that it was not sufficiently long. This was unforeseen given that the half-life of melatonin administered orally is estimated to be approximately 54 min (36). Despite melatonin’s short half-life (37), it might seem that taking it in divided doses per day could provide more even and longer-lasting coverage of its antipruritic and hepatoprotective effects over a 24-hour period. However, it is unclear whether this approach would have required an even longer run-in period. Therefore, while having a carryover effect with a 2-week washout period was a limitation of our study, it appears that administering melatonin once daily was more appropriate in this setting, especially since it mimics the normal physiological secretion pattern of this hormone (38).

## Conclusion

This study demonstrated that a 10-mg daily dose of melatonin is well-tolerated and has significant antipruritic effects on patients with CLD. Moreover, a decrease in the intensity, the extent of affected area and duration of itching, as well as improvements in sleep quality, mood and daily activity levels, were observed in patients who received melatonin. The findings of this research suggest that melatonin may have a promising effect as an antipruritic agent when given as part of the pharmacotherapeutic regimen to patients with liver disease; however, further studies, including larger randomized clinical trials conducted over longer periods, are warranted to confirm these findings.

## Supplementary Materials


