# Supplementary material for: A Pilot Randomized, Clinical Trial of the Anti-pruritus Effect of Melatonin in Patients with Chronic Liver Disease
Source: Iran J Pharm Res. 2021 Spring;20(2):462–72. doi: 10.22037/ijpr.2020.112942.14024 (PMC8457711; doi:10.22037/ijpr.2020.112942.14024)
Supplement: Supplementary file 1 [file ijpr-20-462-s001.pdf]

Supplementary Materials for

A Pilot Randomized, Clinical Trial of the Anti-pruritus Effect of Melatonin in Patients with Chronic Liver Disease

Ayda Esmaeili, Mohssen Nassiri Toosi, Mohammad Taher, Shahin Merat, Jaleh Bayani, Zahra fruzan. Karimian, Aysan Esmaeili, Bobak Moazzami and Soha Namazi

*To whom correspondence should be addressed. E-mail: namazisoha@yahoo.com

Volume 20, Issue 2 (Spring 2021)

This PDF file includes:
Figure S1

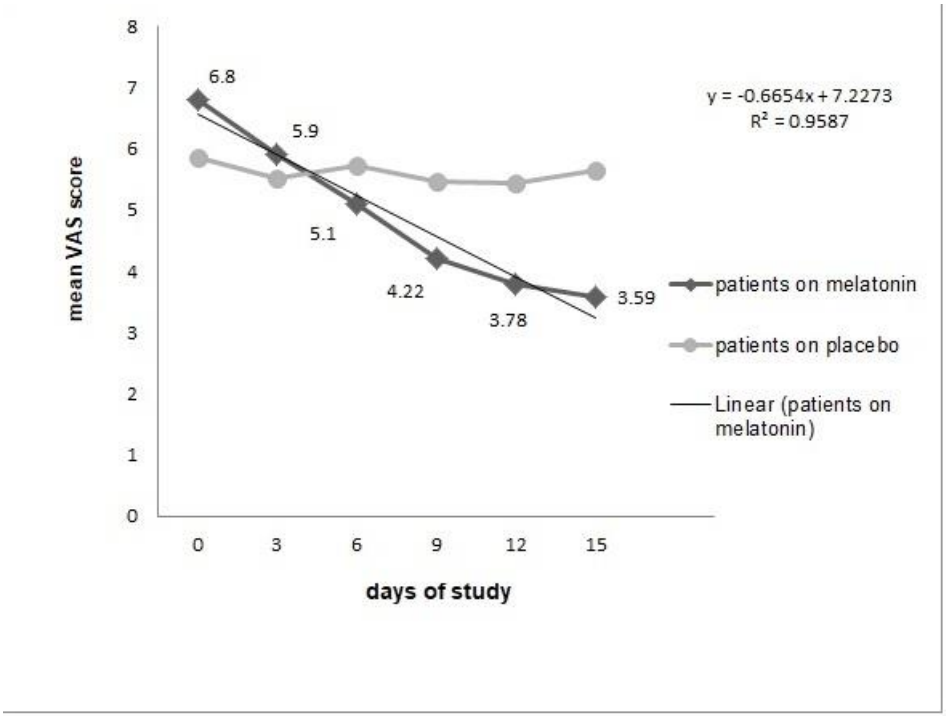

mean VAS score
days of study
y = -0.6654x + 7.2273
R² = 0.9587
6.8
5.9
5.1
4.22
3.78
3.59
patients on melatonin
patients on placebo
Linear (patients on melatonin)

Figure S1.
